# Proton‐Gradient‐Driven Sensitivity Enhancement of Liposome‐Encapsulated Supramolecular Chemosensors

**DOI:** 10.1002/anie.202207950

**Published:** 2022-07-13

**Authors:** Mohamed Nilam, Shreya Karmacharya, Werner M. Nau, Andreas Hennig

**Affiliations:** ^1^ Center for Cellular Nanoanalytics (CellNanOs) and Department of Biology and Chemistry Universität Osnabrück Barbarastraße 7 49069 Osnabrück Germany; ^2^ School of Science Jacobs University Bremen Campus Ring 1 28759 Bremen Germany

**Keywords:** Host–Guest Systems, Liposomes, Macrocycles, pH Gradient, Sensors, Supramolecular Chemistry

## Abstract

An overarching challenge in the development of supramolecular sensor systems is to enhance their sensitivity, which commonly involves the synthesis of refined receptors with increased affinity to the analyte. We show that a dramatic sensitivity increase by 1–2 orders of magnitude can be achieved by encapsulating supramolecular chemosensors inside liposomes and exposing them to a pH gradient across the lipid bilayer membrane. This causes an imbalance of the influx and efflux rates of basic and acidic analytes leading to a significantly increased concentration of the analyte in the liposome interior. The utility of our liposome‐enhanced sensors was demonstrated with various host–dye reporter pairs and sensing mechanisms, and we could easily increase the sensitivity towards multiple biologically relevant analytes, including the neurotransmitters serotonin and tryptamine.

## Graphical abstract

Encapsulating supramolecular chemosensors inside liposomes and exposing them to a pH gradient across the lipid bilayer membrane increases the sensitivity by 1–2 orders of magnitude. The utility of the approach was demonstrated by using various host–dye reporter pairs and sensing mechanisms, which showed increased sensitivity towards multiple biologically relevant analytes, including the neurotransmitters serotonin and tryptamine.

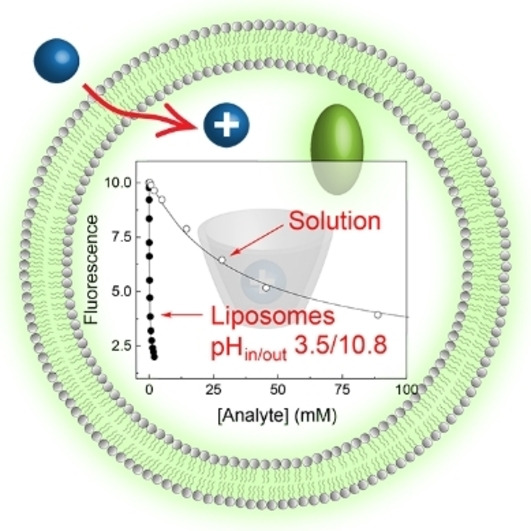

The host–guest inclusion of two or more chemical species integrated together in a facile and reversible manner offers manifold opportunities for the development of novel supramolecular chemosensors.^[1]^ Almost all classes of supramolecular receptors have been utilized for the construction of sensor systems, but the achievable sensitivity is largely limited by the intrinsic binding affinity of the receptor to the analyte. Prototypical supramolecular receptors show frequently only moderate affinities in the μM to mM range, which requires high analyte concentrations to saturate the receptor and produce a sufficient optical output signal. To overcome this limitation, refined receptors with increased affinity generally need to be synthesized.^[2]^ Although astonishing attomolar affinity has been achieved in rare cases for tailored host–guest pairs, such perfect matches have remained limited to cucurbit[7]uril as a synthetic and avidin as a natural receptor.^[3]^ As a remedy, the exploitation of multivalency effects is popular, but this can only be explored for analytes with more than one binding site.^[4]^ Moreover, chemical or enzymatic reactions have been used to selectively convert analytes with low affinity into high affinity guests.^[5]^ However, this approach is limited by the availability of suitable enzymes and chemical reactions, such that the quest for enhanced supramolecular chemosensors is still ongoing.

Herein, we report a broadly applicable approach to enhance the sensitivity of supramolecular chemosensor systems, which could serve as a novel supramolecular liposome‐based sensing platform (Figure 1). The liposome‐enhanced chemosensor system is set up by preparing large unilamellar phospholipid vesicles (LUVs) with supramolecular host–dye reporter pairs encapsulated in the liposome interior. Such vesicles were previously used by us to monitor drug permeation, peptide translocation, and membrane transport through pores.^[6]^ We now additionally apply a transmembrane pH gradient to these LUVs. The idea is that an appropriately applied pH gradient affords external analytes, which are predominantly uncharged and, thus, highly membrane‐permeable, whereas the analyte becomes charged after translocation into the vesicle lumen due to the pH variation. The chemical potential by the pH gradient causes a continuous analyte influx resulting ultimately in a much higher intravesicular analyte concentration than in the surrounding solution. In this manner, a compartmentalized sensing system is set up, where the pH in one compartment is set to facilitate transport to the other compartment, where the pH is optimized for high‐affinity binding to the receptor. At the same time, the compartmentalized influx is continuously driven by a pH gradient, forming a one‐way street for analyte influx to ensure, through a chemical‐potential trick, a highly sensitive detection route. In detail, intravesicular concentrations of analytes can be easily enhanced by 1–2 orders of magnitude, resulting in an equivalent improvement of sensing capability.

**Figure 1:**
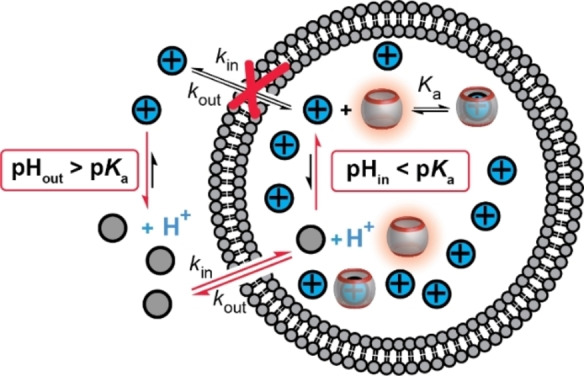
A proton‐gradient‐enhanced compartmentalized sensor system with internalized supramolecular reporters. An externally added, uncharged analyte can permeate through the lipid membrane. Protonation of the analyte in the vesicle lumen renders the analyte membrane‐impermeable. This leads to an overall net influx of the analyte and the strongly increased local concentration affords an amplified response of the sensor system.

Our new sensing concept draws inspiration from an old method to upload drug molecules into liposomes by transmembrane pH gradients,^[7]^ for pre‐concentration in confocal Raman microscopy,^[8]^ or to monitor supramolecular ion transport.^[9]^ Liposomes are also largely compatible with conventional biosensors,^[10]^ but the use of transmembrane pH gradients has so far been unexplored in the context of sensor systems.^[11]^ We demonstrate the broad applicability of our approach with a representative set of model analytes comprising, e.g. the neurotransmitters tryptamine and serotonin, the Parkinson's drug amantadine, or the biogenic amine putrescine, and we show that the sensitivity of liposome‐encapsulated supramolecular reporter pairs based on cyclodextrins (CDs) and cucurbiturils (CBs) is significantly enhanced in the presence of a suitable pH gradient (Figure 2).

**Figure 2:**
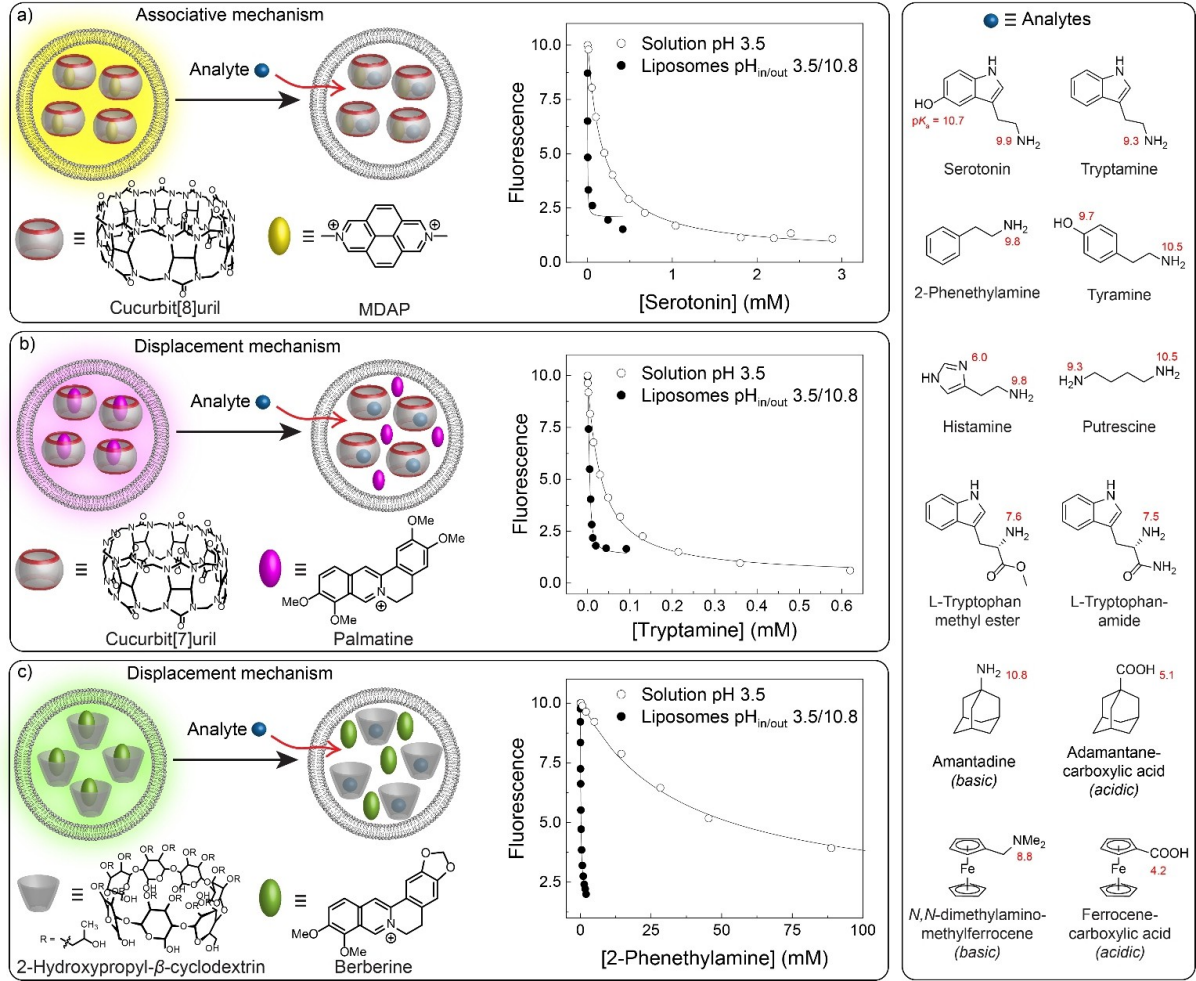
Enhanced sensitivity of liposome‐encapsulated reporter pairs for the detection of a) serotonin (with CB8/MDAP: *λ*_ex_=338 nm and *λ*_em_=423 nm), b) tryptamine (with CB7/PLM: *λ*_ex_=425 nm and *λ*_em_=495 nm), and c) 2‐phenethylamine (with HP‐β‐CD/BE: *λ*_ex_=420 nm and *λ*_em_=540 nm). The liposomes were prepared with 100 mM Na citrate, pH 3.5 inside, and 100 mM Na_2_HPO_4_, pH 10.8 outside. The titration plots with liposomes (filled circles) were obtained by monitoring the time‐dependent fluorescence during successive addition of the analytes and the fluorescence intensities after equilibration were plotted against the concentration of added analyte (see Supporting Information for details). For comparison the response in 100 mM Na citrate, pH 3.5, without liposomes is shown (open circles). In the analyte chart, p*K*_a_ values are given in red.

As a first piece of demonstration, we selected cucurbit[8]uril (CB8) and 2,7‐dimethyldiazapyrenium (MDAP) as a liposome‐encapsulated reporter pair and the neurotransmitter serotonin as an analyte (Figure 2a). CB8 forms ternary complexes with the fluorescent dye MDAP and electron‐rich, aromatic guests, in which the fluorescence is quenched. This can be used for sensing and has been termed an associative binding assay.[^6a^, ^6c^, ^6d^, ^12^] In good accordance with literature^[12]^ we find a binding constant of *K*_a_=5.7×10^3^ M^−1^ for serotonin in a conventional fluorescence titration (without liposomes). As a consequence of this low affinity, ca. 200 μM serotonin are required to afford 50 % of the maximal signal change of the CB8/MDAP chemosensor, which was strongly reduced, to 7.5 μM, in the proton‐gradient‐driven, liposome‐enhanced variant with an internal pH of 3.5 and an external pH of 10.8.

An internal pH of 3.5 was chosen to ensure protonation of all analytes, whereas the external pH of 10.8 was chosen to deprotonate the amino group (p*K*_a_=9.9, >80 % deprotonated) but not too much of the phenolic OH group (p*K*_a_=10.7, ca. 50 % deprotonated, see Figures 2d and Table S3 for p*K*_a_ values). Using these conditions, serotonin is sufficiently membrane‐permeable to fully equilibrate its inside and outside concentrations within minutes and to produce a stable fluorescence intensity (Figure S5). Fitting of the fluorescence intensity gave an apparent binding constant of *K*_app_=7.7×10^5^ M^−1^ (Figure 2a, filled circles). The liposome‐encapsulated CB8/MDAP reporter pair thus provides a much higher sensitivity (enhancement factor *E*_f_=135) enabling serotonin detection in the low μM concentration range. This principle could be easily transferred to other analytes and enabled the straightforward nanomolar detection of, for example, tryptamine (Figures S6–S9 and Table 1). The sensitivity enhancement was similarly good in the presence of blood serum (Figures S10), which improved the limit of detection (LOD) of serotonin by the CB8/MDAP chemosensor from 120 μM to 1.6 μM in spiked blood serum samples (Figures S11, S12).

**Table 1:** Binding affinities of different reporter pairs to different analytes in homogeneous solution and apparent affinities of the analytes towards the different hosts in liposomes with a pH gradient.

Reporter pair	Analyte	*K*_a_ [M^−1^] solution^[a]^	*K*_app_ [M^−1^] liposomes^[b]^	***E***_**f**_^**[c]**^
CB8/MDAP	Serotonin^[d]^	5.7×10^3^ (2.5×10^3^)	7.7×10^5^ (1.3×10^5^)	**135** (52)
	Tryptamine	9.9×10^4^	1.2×10^7^	**121**
	Tyramine	6.0×10^3^	5.3×10^5^	**88**
	L‐Tryptophanamide	2.3×10^5^	1.8×10^7^	**78**
	L‐Tryptophan methyl ester	1.3×10^5^	3.5×10^6^	**27**
CB7/PLM	Tryptamine^[d]^	3.2×10^4^ (1.9×10^4^)	2.5×10^5^ (1.9×10^5^)	**8.0** (10)
	2‐Phenethylamine	4.6×10^6^	2.3×10^7^	**5.0**
	Tyramine	1.0×10^6^	3.1×10^6^	**3.0**
	Putrescine	1.0×10^5^	2.0×10^6^	**20**
	Histamine	1.1×10^4^	2.0×10^5^	**18**
HP‐β‐CD/ BE	2‐Phenethylamine	29	7.4×10^3^	**255**
	1‐Adamantylamine	2.8×10^4^	1.6×10^6^	**57**
	*N,N‐*dimethylamino‐ methylferrocene	3.2×10^3^	5.2×10^5^	**162**
	1‐Adamantane‐ carboxylic acid^[e]^	3.8×10^4^	7.2×10^6^	**189**
	Ferrocenecarboxylic acid^[e]^	3.6×10^4^	1.1×10^6^	**31**

[a] The binding constants in solution were measured using the same buffer as that for the liposome‐enhanced measurement. Error ca. 10 %. [b] Apparent binding constants for liposome‐encapsulated reporter pairs exposed to a pH gradient. Error ca. 20 %. Unless noted otherwise, an internal pH of 3.5 and an external pH of 10.8 was used. [c] Enhancement factor *E*_f_=*K*_app_/*K*_a_. [d] The apparent affinities in the presence of 5 % blood serum are given in brackets. [e] The carboxylic acid analytes were measured with an internal pH of 10.8 and an external pH of 3.0.

Next, we investigated liposome‐encapsulated reporter pairs exposed to a pH gradient by means of an intravesicular indicator displacement assay (Figure 2b). We initially utilized the established reporter pair cucurbit[7]uril (CB7) and berberine (BE),[^6a^, ^6d^, ^6e^] which showed, however, a reduced sensitivity to the target analytes (Figures S14, S15 and Table S2). This is most likely due to the much higher intravesicular concentrations of CB7 and BE compared to the bulk solution. Considering that, with the premise of quantitative complexation conditions, 50 % displacement is achieved, when the products of the binding constants and concentrations of the dye and competitor are equal ([HC]=[HD] when [C]_tot_ *K*_a,C_=[D]_tot_ *K*_a,dye_),^[13]^ this would mean that ca. 120 mM intravesicular tryptamine is required to displace 50 % of intravesicular BE. It is interesting to note that this consideration provides indirect evidence that the proton‐gradient‐driven intravesicular enrichment of the analyte was still operative with CB7/BE, because only ca. 1 mM externally added tryptamine was required to efficiently compete with the nanomolar affinity of the CB7/BE reporter pair inside the vesicle; however, the CB7/BE reporter pair in solution was still more sensitive.

In order to enhance the sensitivity beyond that of the reporter pair in homogeneous solution, a host–dye reporter pair with a much lower binding affinity was therefore required, and we considered CB7 and palmatine (CB7/PLM) as a suitable alternative.^[14]^ The binding affinity of CB7/PLM is much lower (4.3×10^4^ M^−1^) than that of CB7/BE; it can also be stably encapsulated in liposomes (see Supporting Information), and it gave indeed the expected increase in sensitivity (Figures S16–S21 and Table 1). For example, the affinity of tryptamine increased from *K*_a_=3.2×10^4^ M^−1^ in homogeneous solution to an apparent affinity of *K*_app_=2.5×10^5^ M^−1^ with the liposome‐enhanced CB7/PLM reporter pair. Consequently, the amount to displace 50 % of PLM from CB7 was lowered from 40 μM to 5 μM tryptamine (Figure 2b). The enhancement factors for the CB7/PLM reporter pair were overall lower than for the CB8/MDAP reporter pair (Table 1), which could, however, be principally accounted for with reporter pairs that possess even lower host–dye affinities or optimized intravesicular reporter pair concentrations. Also here, transferability of the concept to tryptamine concentration determinations in blood serum was unproblematic, and the LOD improved from 17 μM to 1.2 μM (Figures S22–S24).

To also demonstrate the transferability of our approach to another class of host molecules, cyclodextrins were selected, which are infamous for their rather limited binding affinities.^[15]^ As a test case, 2‐hydroxypropyl‐β‐cyclodextrin (HP‐β‐CD) and BE were established as a liposome‐encapsulated host–dye reporter pair. The binding constant between HP‐β‐CD and BE was determined as *K*_a_=(137±4) M^−1^ (Figure S25) and the HP‐β‐CD/BE reporter pair was successfully encapsulated to afford stable liposomes with a diameter of ca. 170 nm (see Supporting Information). β‐CD and its derivatives are known to bind various small hydrophobic analytes with typical affinities in the range from 10^1^ to 10^4^ M^−1^.^[15]^ This was also found for our selection of analytes (Table 1), which all required micromolar or millimolar analyte concentrations to afford a sensor response in homogeneous solution. In contrast, a significant increase in sensitivity was observed for all analytes with the pH gradient‐driven, liposome‐encapsulated HP‐β‐CD/BE reporter pair (see Table 1 and Figures S26–S28). Specifically, the binding constant of phenethylamine was only 29 M^−1^ in homogeneous solution, requiring high millimolar concentrations to affect an optical response, whereas the sensitivity increased ca. 250‐fold with the liposome‐enhanced sensor system, enabling detection of micromolar concentrations of phenethylamine (Figure 2c).

Finally, the concept was also tested with analytes containing carboxylic acid groups. In this “pH‐inverted” case, HP‐β‐CD/BE liposomes were prepared with a low external pH, at which the carboxylic acid group is protonated and membrane‐permeable, whereas the internal pH was sufficiently high to maintain the analytes in their membrane‐impermeable carboxylate form. Ferrocenecarboxylic acid and 1‐adamantanecarboxylic acid were selected as model analytes, which both showed an increased sensitivity by a factor of ca. 30 and 190 with the inverse proton‐gradient‐driven, liposome‐enhanced sensor system (Table 1 and Figure S29, S30). As a particularly noteworthy asset, HP‐β‐CD/BE liposomes with an outside acidic pH and an inside basic pH did not respond to amantadine at all (Figure 3a) suggesting that an appropriately applied pH gradient cannot only be used to increase the sensitivity, but also the selectivity of liposome‐encapsulated reporter pairs. By rational inversion of the pH gradient, the liposome‐encapsulated HP‐β‐CD/BE becomes either selective for adamantane carboxylic acid (Figure 3a) or amantadine (Figure 3b), whereas both analytes were indistinguishable in homogeneous solution. This provides an extended application of the reporter‐pair‐based membrane assay principle; supramolecular receptors are not only encapsulated inside liposomes in order to obtain 1–2 orders of magnitude sensitivity enhancement through a pH gradient, but the membrane additionally acts as a selector or gate on the basis of the pH‐dependent permeabilities of analytes.

**Figure 3:**
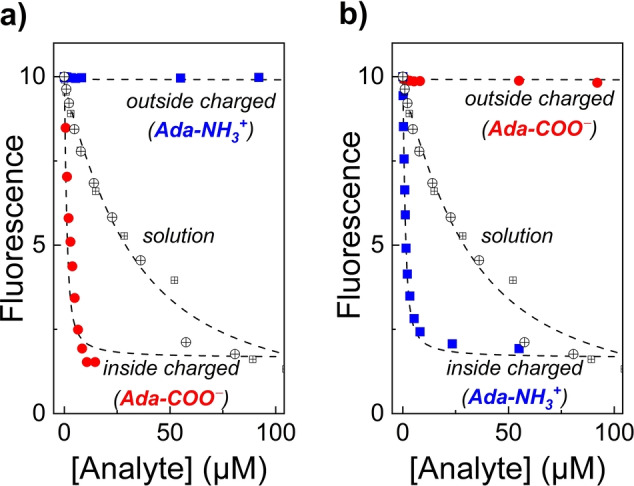
Discrimination of adamantane carboxylic acid (red circles) and amantadine (blue squares) by HP‐β‐CD/BE liposomes exposed to a) a pH_in/out_=10.8/3.0 gradient and b) a pH_in/out_=3.5/10.8 gradient. The response of the charged analytes in homogeneous solution is shown in both panels for comparison.

Overall, we have shown herein that the sensitivity of supramolecular chemosensors can be significantly enhanced by microencapsulation into liposomes with a pH gradient that renders the external analytes uncharged and permeable, whereas the analytes become charged and impermeable after entering the vesicle lumen. This is in accordance with an enrichment of the analytes inside the vesicles leading to strongly increased intravesicular concentrations compared to the surrounding solution.[^7b^, ^8a^] This method enables the sensitive detection of analytes with low binding affinity to supramolecular chemosensors. In future work, the presented concept may also be extended to other mechanisms that affect the membrane permeability of analytes than protonation or deprotonation, e.g., to enzymatic or chemical transformations. Our results further suggest that host–dye combinations, which were previously disregarded as reporter pairs due to their low affinity,^[16]^ can be revived as functional chemosensors when encapsulated in liposomes; in fact, the low‐affinity reporter pairs responded more sensitively than established reporter pairs, which provides new avenues for the application of low‐affinity dyes in supramolecular sensor systems. Finally, we have also shown that different pH gradients afford a differential response of the liposome‐encapsulated reporter pairs towards otherwise indistinguishable analytes. This may be useful in the construction of differential sensor arrays, which is a typical approach to improve the selectivity of supramolecular chemosensors in detecting specific analytes.^[1a]^

## Conflict of interest

The authors declare no conflict of interest.

## Supporting information

As a service to our authors and readers, this journal provides supporting information supplied by the authors. Such materials are peer reviewed and may be re‐organized for online delivery, but are not copy‐edited or typeset. Technical support issues arising from supporting information (other than missing files) should be addressed to the authors.

Supporting Information

## Data Availability

The data that support the findings of this study are available in the Supporting Information of this article.
