# Supplementary material for: Proton‐Gradient‐Driven Sensitivity Enhancement of Liposome‐Encapsulated Supramolecular Chemosensors
Source: Angew Chem Int Ed Engl. 2022 Jul 13;61(35):e202207950. doi: 10.1002/anie.202207950 (PMC9543936; doi:10.1002/anie.202207950)
Supplement: Supplementary file 1 — Supporting Information [file ANIE-61-0-s001.pdf]

Angewandte Chemie
Eine Zeitschrift der Gesellschaft Deutscher Chemiker

Supporting Information

Proton-Gradient-Driven Sensitivity Enhancement of Liposome-Encapsulated Supramolecular Chemosensors

M. Nilam, S. Karmacharya, W. M. Nau, A. Hennig**

Supporting Information

Table of Contents

Materials and Methods 2
Materials 2
Instruments 2
Liposome Preparations 3
Liposome Characterization 4
Determination of Binding Constants 6
Measurements with Blood Serum Samples 7
Supporting Results 8
CB8/MDAP Reporter Pair 8
CB7/BE Reporter Pair 12
CB7/PLM Reporter Pair 14
HP-β-CD/BE Reporter Pair 19
Buffer Capacity 23
References 25

Materials and Methods

Materials

1-Palmitoyl-2-oleoyl-*sn*-glycero-3-phosphocholine (POPC) and 1-palmitoyl-2-oleoyl-*sn*-glycero-3-phospho-L-serine (POPS) were purchased from Avanti Polar Lipids (Alabaster, AL, USA). Cucurbit[7]uril (CB7) and cucurbit[8]uril (CB8) were purchased from Strem Chemicals (Kehl, Germany) or synthesized according to the previous literature.[1] The fluorescent dye 2,7-dimethyldiazapyrenium (MDAP) was synthesized according to the literature.[2] Tryptamine hydrochloride, tryptophan methyl ester hydrochloride (Trp-OMe), L-tryptophanamide hydrochloride (Trp-NH_2), serotonin hydrochloride, tyramine hydrochloride, 2-phenylethylamine hydrochloride, putrescine dihydrochloride, histamine dihydrochloride, amantadine hydrochloride, 1-adamantanecarboxylic acid (ADA-COOH), berberine chloride (BE), palmatine chloride (PLM), and 8-hydroxypyrene-1,3,6-trisulfonate trisodium salt (HPTS) were purchased from Sigma-Aldrich (Steinheim, Germany). Blood serum (human male AB) was from Sigma-Aldrich (Steinheim, Germany). NAP-25 columns (Sephadex G-25 DNA grade) were purchased from GE Healthcare (Buckinghamshire, UK). *N,N*-dimethylaminomethylferrocene (FC-NH_2) and ferrocenecarboxylic acid (FC-COOH) were purchased from Alfa Aesar (Massachusetts, USA), and (2-hydroxypropyl)-β-cyclodextrin (HP-β-CD) was obtained from CycloLab (Budapest, Hungary).

Instruments

Dynamic light scattering (DLS) measurements were performed with a Zetasizer Nano from Malvern Instruments and fluorescence measurements were performed in quartz glass cuvettes with a Varian Cary Eclipse spectrofluorometer or a Jasco FP-8300 spectrofluorometer equipped with temperature-controlled stirrers.

Buffer Preparations

All buffers were prepared by dissolving the acid form of the buffer and addition of NaOH to adjust the desired pH. For example, the sodium citrate buffers were prepared from citric acid, the 100 mM NaH_2PO_4, pH 7.5 buffer from sodium dihydrogen phosphate and the 100 mM Na_2HPO_4, pH 10.8 buffer from disodium hydrogen phosphate with subsequent addition of NaOH to adjust the pH.

Liposome Preparations

CB8/MDAP liposomes (POPC/POPS⊃CB8/MDAP). 100 µL 25 mg/ml POPC and 33 µL 10 mg/ml POPS in chloroform were mixed in a 5-mL round bottom flask and purged with nitrogen to obtain a thin lipid film. The film was dried overnight under high vacuum and rehydrated with 1 mL rehydration buffer (0.5 mM CB8 and 0.55 mM MDAP in either 100 mM Hepes, pH 7.5 or 100 mM sodium citrate, pH 3.5). After gentle agitation at ambient temperature for 30 minutes, the liposome suspensions were subjected to 15 freeze-thaw cycles. The external buffer was subsequently exchanged by size exclusion chromatography (NAP 25) with 100 mM Na_2HPO_4, pH 10.8.

CB7/BE liposomes (POPC/POPS⊃CB7/BE). 100 µL 25 mg/ml POPC and 33 µL 10 mg/ml POPS in chloroform were mixed in a 5-mL round bottom flask and purged with nitrogen to obtain a thin lipid film. The film was dried overnight under high vacuum and rehydrated with 1 mL 100 mM sodium citrate, 0.3 mM CB7, 0.5 mM BE, pH 3.5. After gentle agitation at ambient temperature for 30 minutes, the liposome suspensions were subjected to 15 freeze-thaw cycles. The external buffer was subsequently exchanged by size exclusion chromatography (NAP 25) with 100 mM Na_2HPO_4, pH 10.8.

CB7/PLM liposomes (POPC/POPS⊃CB7/PLM). 100 µL 25 mg/ml POPC and 33 µL 10 mg/ml POPS in chloroform were mixed in a 5-mL round bottom flask and purged with nitrogen to obtain a thin lipid film. The film was dried overnight under high vacuum and rehydrated with 1 mL rehydration buffer (1 mM CB7 and 1 mM PLM in either 100 mM NaH_2PO_4, pH 7.5 or 100 mM sodium citrate, pH 3.5). After gentle agitation at ambient temperature for 30 minutes, the liposome suspensions were subjected to 15 freeze-thaw cycles. The external buffer was subsequently exchanged by size exclusion chromatography (NAP 25) with 100 mM Na_2HPO_4, pH 10.8.

HP-*β*-CD/BE liposomes (POPC/POPS⊃HP-*β*-CD/BE). 200 µL 25 mg/ml POPC and 66 µL 10 mg/ml POPS in chloroform were mixed in a 5-mL round bottom flask and purged with nitrogen to obtain a thin lipid film. The film was dried overnight under high vacuum and rehydrated with 1 mL rehydration buffer (20 mM HP-*β*-CD and 1 mM BE in either 100 mM NaH_2PO_4, pH 7.5, 100 mM sodium citrate, pH 3.5, or 100 mM Na_2HPO_4, pH 10.8). After gentle agitation at ambient temperature for 30 minutes, the liposome suspensions were subjected to 15 freeze-thaw cycles. The external buffer was subsequently exchanged by size exclusion chromatography (NAP 25) with either 100 mM Na_2HPO_4, pH 10.8 or 100 mM sodium citrate, pH 3.0.

HPTS liposomes (POPC/POPS⊃HPTS). 200 µL 25 mg/ml POPC 66 µL 10 mg/ml POPS in chloroform were mixed in a 5-mL round bottom flask and purged with nitrogen to obtain a thin lipid film. The film was dried overnight under high vacuum and rehydrated with 1 mL rehydration buffer (1 mM HPTS 100 mM NaH_2PO_4, pH 7.2). After gentle agitation at ambient temperature for 30 minutes, the liposome suspensions were subjected to 15 freeze-thaw cycles. The external buffer was subsequently exchanged by size exclusion chromatography (NAP 25) with 100 mM Na_2HPO_4, pH 10.8.

Liposome Characterization

The concentrations of the phospholipids in the resulting liposome stock solutions was determined by our NMR method[3] and the size of the liposomes was determined by DLS. The total phospholipid concentrations were ca. 27 μM for experiments with the CB8/MDAP, CB7/BE, and CB7/PLM reporter pairs and ca. 55 μM for the HP-β-CD/BE reporter pair. For all liposome preparations, an unimodal size distribution was observed (Fig. S1) and the size of all liposomes was in the range of ca. 150-200 nm (Table S1).

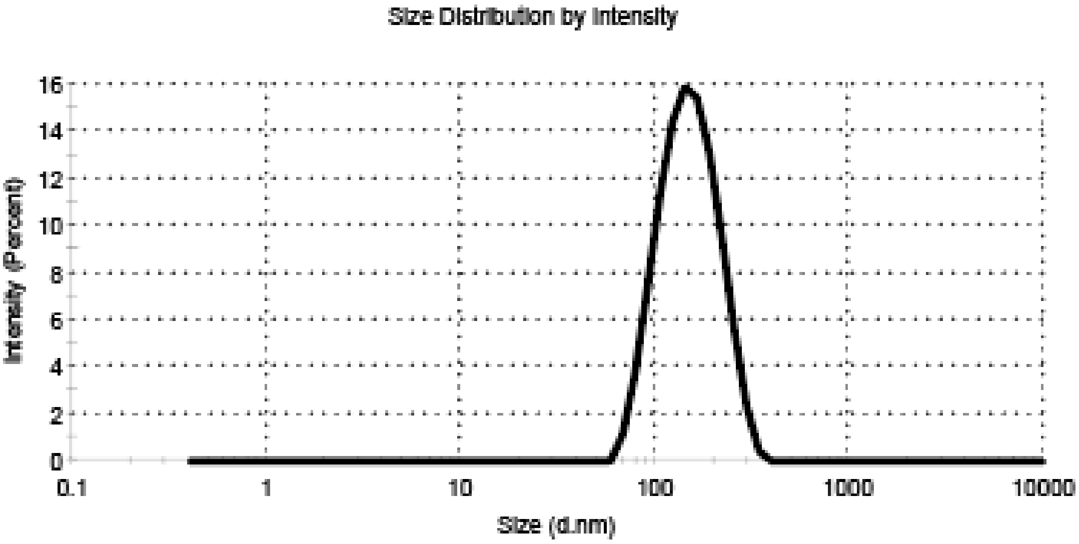

Figure S1. Size distribution of POPC/POPS⊃CB7/PLM vesicles by dynamic light scattering (DLS, d = 139 nm).

Table S1. Hydrodynamic diameter of liposomes measured by DLS.

Liposome	inside pH	outside pH	Diameter (nm)
CB8/MDAP	3.5	10.8	145 ± 2.0
	7.5	10.8	134 ± 1.0
CB7/BE	3.5	10.8	141 ± 1.0
CB7/PLM	3.5	10.8	139 ± 1.0
	7.5	10.8	143 ± 1.0
HP-*β*-CD/BE	3.5	10.8	163 ± 2.0
	7.5	10.8	178 ± 2.0
	7.5	3.0	147 ± 1.0
	10.8	3.0	172 ± 1.0

Liposome Stability

The experiments were usually performed within four days after liposome preparation. During that time, no alterations in the liposome size or in the performance of the liposome-encapsulated reporter pairs was noted. Also, the fluorescence intensity of the liposomes did not change significantly (Fig. S2).

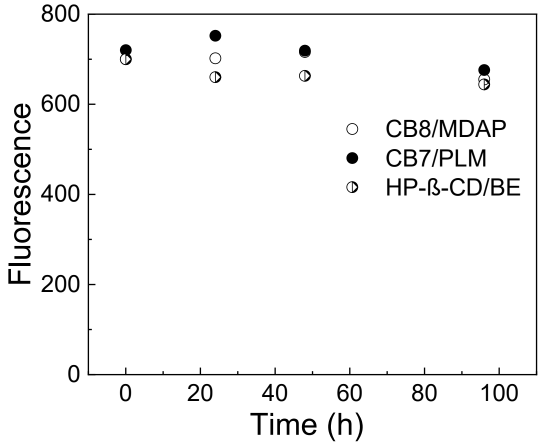

Figure S2. Fluorescence intensity of liposomes with encapsulated reporter pairs over a time period of up to 96 hours. The internal pH of all liposomes was pH 3.5 and the external pH was pH 10.8.

pH Gradient Stability

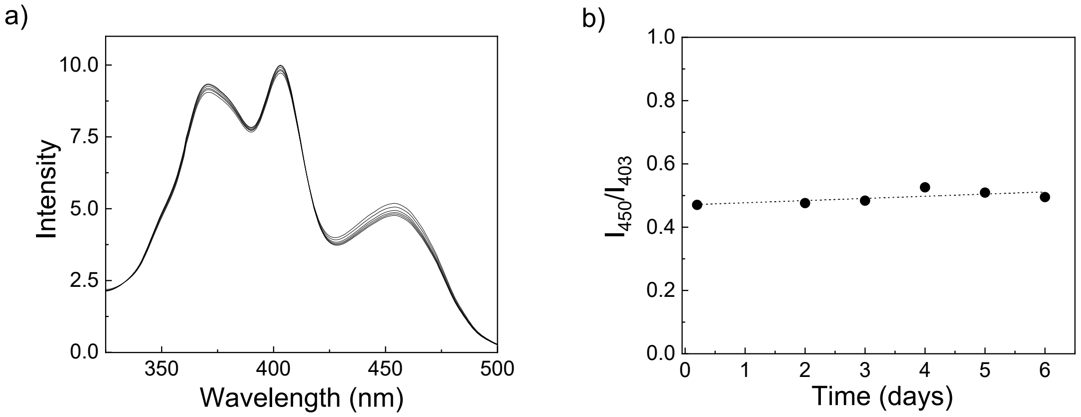

Figure S3. a) Excitation spectra (λ_{em} = 511 nm) of HPTS-encapsulated POPC/POPS liposomes (inside: 100 mM NaH_2PO_4, 1 mM HPTS, pH 7.2; outside: 100 mM Na_2HPO_4, pH 10.8). b) Ratiometric signal of HPTS-encapsulated liposomes as a measure of internal pH over a time period of up to 6 days.

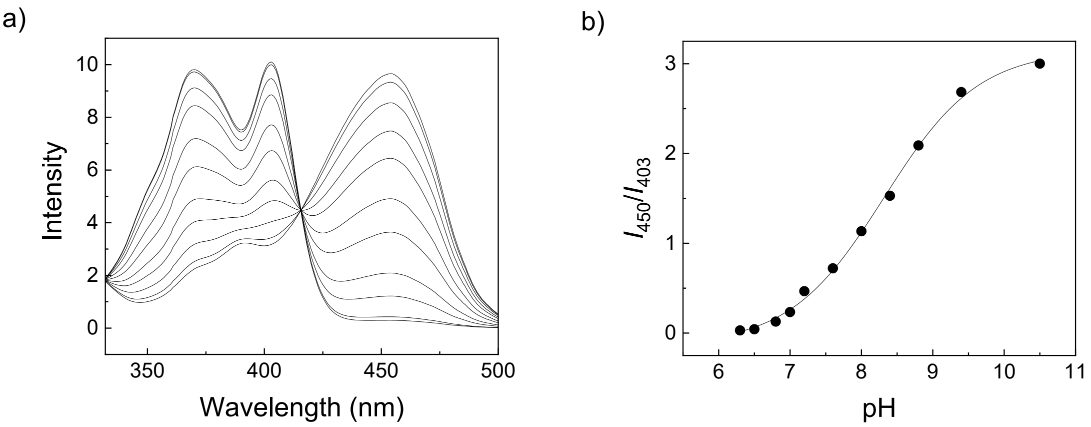

Figure S4. a) pH dependence of the excitation spectrum (λ_{em} = 511 nm) of 1 μM HPTS in 100 mM sodium phosphate buffer. b). Plot of ratiometric fluorescence intensity (I_{450}/I_{403}) against pH.

Determination of Binding Constants

Titrations in Homogeneous Solution

Conventional fluorescence titrations were performed and the data was analyzed as previously described.[4]

Titrations with Liposome-Encapsulated Reporter Pairs

Titrations with the liposome-encapsulated reporter pairs were performed by measuring the time-dependent fluorescence changes of the reporter pairs at suitable excitation and emission wavelengths (CB8/MDAP: λ_{ex} = 338 nm, λ_{em} = 423 nm; CB7/BE: λ_{ex} = 420 nm, λ_{em} = 495 nm; CB7/PLM: λ_{ex} = 425 nm, λ_{em} = 495 nm; HP-β-CD/BE: λ_{ex} = 420 nm, λ_{em} = 540 nm). Therefore, 20 μL of reporter pair-encapsulated liposomes were diluted with 1980 μL 100 mM Na_2HPO_4, pH 10.8 for basic analytes or 100 mM sodium citrate, pH 3.0 for acidic analytes in fluorescence quartz glass cuvettes. Fluorescence was then monitored continuously during successive addition of the analytes, whereas the sample was allowed to fully equilibrate inside and outside concentrations after each addition as indicated by a constant fluorescence intensity. The total volume of added analyte stock solution did not exceed 100 μL (<5%), such that fluorescence intensity changes originating from dilution were not corrected. The constant fluorescence intensity after equilibration was then plotted against the total concentration of added analyte and the data was analyzed as previously described (assuming homogeneous solution conditions, see next paragraph).

Comparability of Measurements in Liposomes and Homogeneous Solution

To afford a reliable comparison of the fluorescence changes and the resulting apparent and true binding constants of the analytes with liposome-encapsulated reporter pairs and reporter pairs in homogeneous solution, it is most desirable that the reporter pair concentrations are below the dissociation constant, K_d, of the analyte. In this case, the shape of the fluorescence response curves become independent of the reporter pair concentrations, whereas at reporter pair concentrations much above the K_d of the analyte, the response of the sensor would depend on the reporter pair concentrations. As an extreme example, quantitative binding results at concentrations much above the K_d, such that full displacement (100% response) occurs when $[host]_{tot}$ = $[analyte]_{tot}$. It was thus ensured that the concentrations of the reporter pair concentrations were below the K_d of the analyte in solution for all investigated combinations (except for 2-phenethylamine with CB7/PLM at pH 3.5). Additionally, the concentrations of the liposome-encapsulated reporter pairs were always below the reporter pair concentrations in homogeneous solution to ensure that an overlooked dependence on the reporter pair concentration would rather lead to a decreased than to increased sensitivity of the liposome-encapsulated reporter pairs.

Measurements with Blood Serum Samples

Apparent Binding Constants

Apparent binding constants in 5% blood serum were measured by adding 20 µL of reporter pair-encapsulated liposomes to 1880 µL of the respective buffer (see above), 100 µL blood serum and successive addition of varying amounts of analyte. After each addition, the sample was allowed to fully equilibrate inside and outside concentrations and the constant fluorescence intensity was plotted and analyzed as described above. In homogeneous solution, 100 µL were added to 1900 µL buffer containing the reporter pair and titrations were conducted and analyzed as described above.

LOD Determinations

Spiked blood serum samples were prepared by adding known amounts of the analyte from a highly concentrated stock solution to human blood serum such that dilution effects of the blood serum can be neglected. 100 µL of the spiked blood serum samples were added to 1900 µL reporter pair (either liposome-encapsulated or in solution) and the fluorescence was recorded until a constant value was reached. The constant fluorescence intensity was then plotted against the analyte concentration the spiked blood serum samples. LOD values were then calculated from the slope and standard deviation of the linearly fitted line.

Supporting Results

CB8/MDAP Reporter Pair

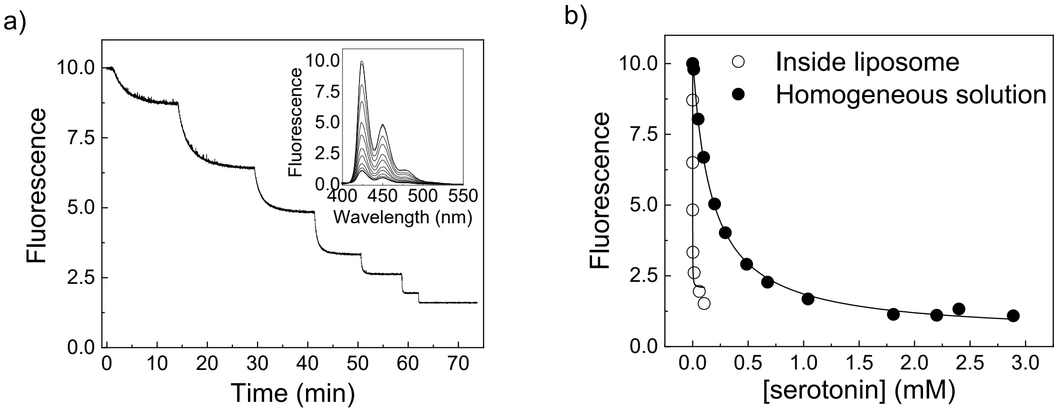

Figure S5. a) Time-dependent fluorescence changes of POPC/POPS⊃CB8/MDAP liposomes (pH_{out} = 10.8, pH_{in} = 3.5) during successive addition of serotonin. The inset shows the fluorescence spectral changes during a conventional fluorescence titration with serotonin and CB8/MDAP (0.5 μM CB8; 0.55 μM MDAP) in 100 mM sodium citrate, pH 3.5. b) Respective titration curves (λ_{ex} = 338 nm and λ_{em} = 423 nm).

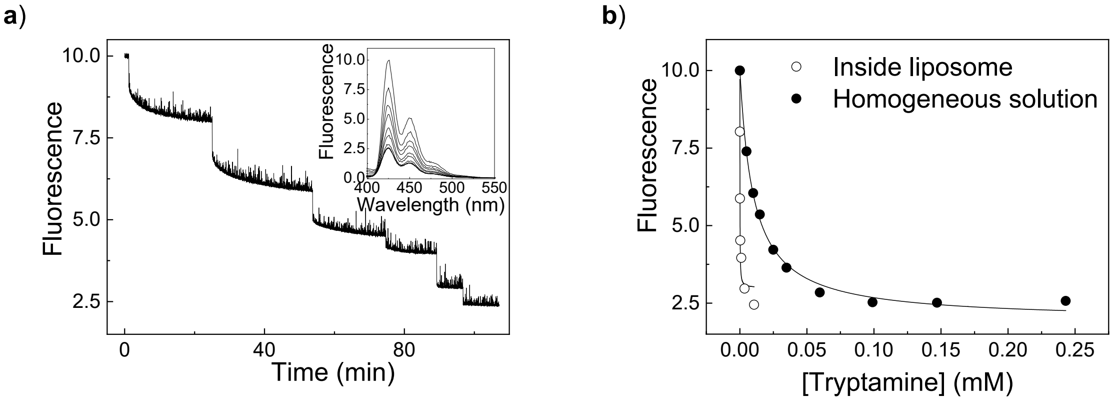

Figure S6. a) Time-dependent fluorescence changes of POPC/POPS⊃CB8/MDAP liposomes (pH_{out} = 10.8, pH_{in} = 3.5) during successive addition of tryptamine. The inset shows the fluorescence spectral changes during a conventional fluorescence titration with tryptamine and CB8/MDAP (0.5 μM CB8; 0.55 μM MDAP) in 100 mM sodium citrate, pH 3.5. b) Respective titration curves (λ_{ex} = 338 nm and λ_{em} = 423 nm).

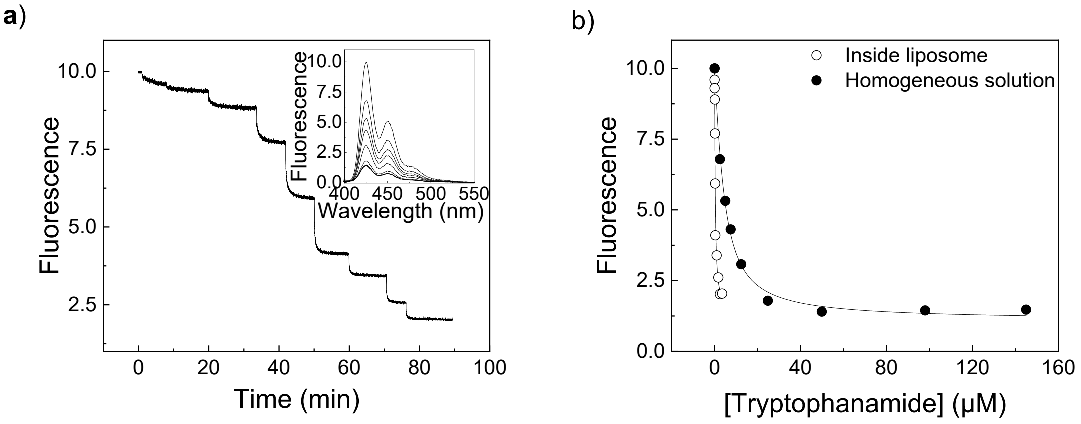

Figure S7. a) Time-dependent fluorescence changes of POPC/POPS⊃CB8/MDAP liposomes (pH_{out} = 10.8, pH_{in} = 3.5) during successive addition of tryptophanamide. The inset shows the fluorescence spectral changes during a conventional fluorescence titration with tryptophanamide and CB8/MDAP (0.5 μM CB8; 0.55 μM MDAP) in 100 mM sodium citrate, pH 3.5. b) Respective titration curves (λ_{ex} = 338 nm and λ_{em} = 423 nm).

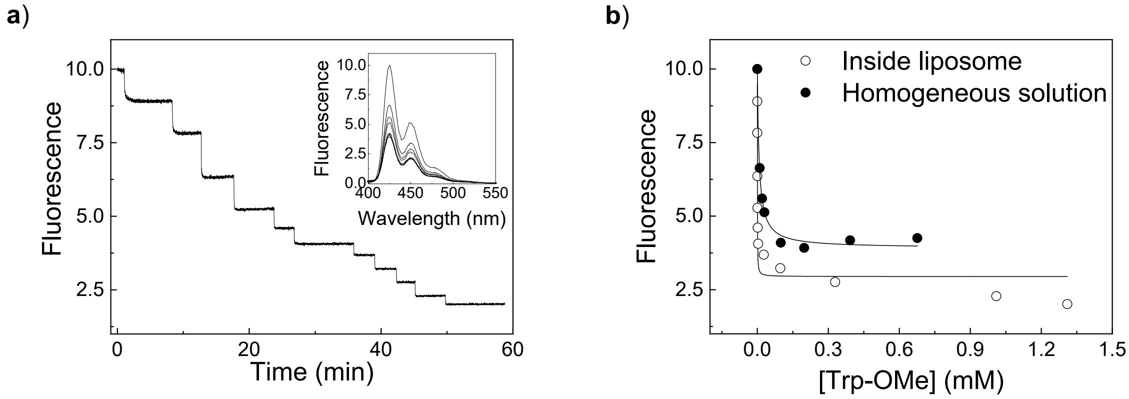

Figure S8. a) Time-dependent fluorescence changes of POPC/POPS⊃CB8/MDAP liposomes (pH_{out} = 10.8, pH_{in} = 3.5) during successive addition of tryptophan methyl ester. The inset shows the fluorescence spectral changes during a conventional fluorescence titration with tryptophan methyl ester and CB8/MDAP (0.5 μM CB8; 0.55 μM MDAP) in 100 mM sodium citrate, pH 3.5. b) Respective titration curves (λ_{ex} = 338 nm and λ_{em} = 423 nm).

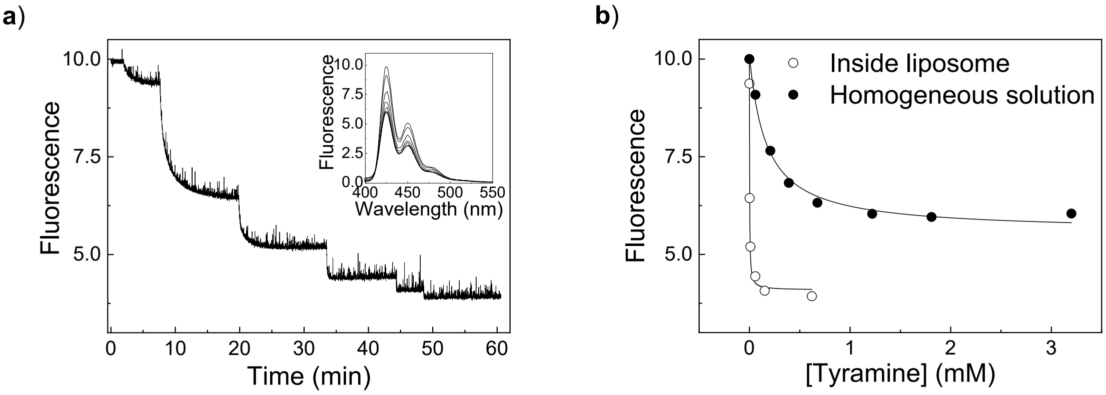

Figure S9. a) Time-dependent fluorescence changes of POPC/POPS⊃CB8/MDAP liposomes (pH_{out} = 10.8, pH_{in} = 3.5) during successive addition of tyramine. The inset shows the fluorescence spectral changes during a conventional fluorescence titration with tyramine and CB8/MDAP (0.5 μM CB8; 0.55 μM MDAP) in 100 mM sodium citrate, pH 3.5. b) Respective titration curves (λ_{ex} = 338 nm and λ_{em} = 423 nm).

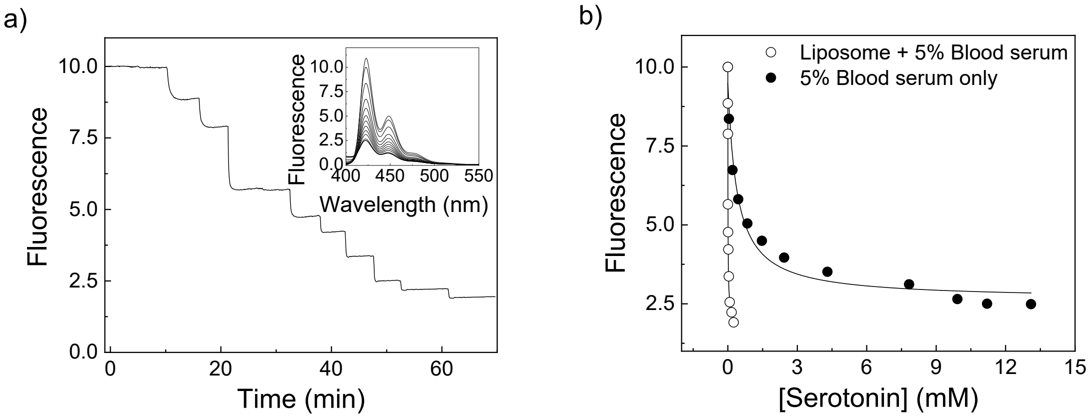

Figure S10. a) Time-dependent fluorescence changes of POPC/POPS⊃CB8/MDAP liposomes (pH_{out} = 10.8, pH_{in} = 3.5) during successive addition of serotonin in presence of 5% blood serum. The inset shows the fluorescence spectral changes during a conventional fluorescence titration with serotonin and CB8/MDAP (0.5 μM CB8; 0.55 μM MDAP) in 100 mM sodium citrate, pH 3.5, 5% blood serum. b) Respective titration curves (λ_{ex} = 338 nm and λ_{em} = 423 nm).

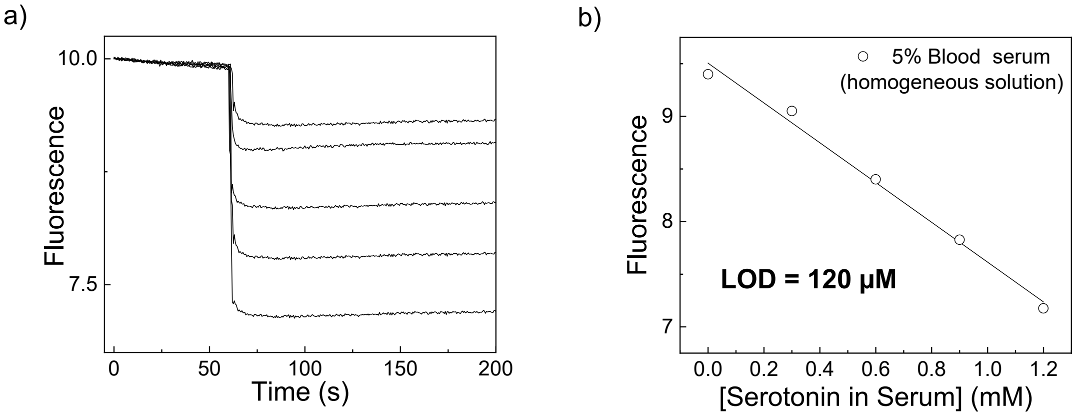

Figure S11. a) Fluorescence changes of CB8/MDAP (0.5 μM CB8 and 0.55 μM MDAP) upon addition of 5% human blood serum spiked with varying concentration of serotonin in 100 mM sodium citrate, pH 3.5 b) Plot of the final fluorescence at ca. 200 s against the serotonin concentration in the spiked blood serum samples. The limit of detection (LOD) of serotonin was calculated from the slope and the triple standard deviation of the blank of the linear calibration curve ($R^2 = 0.99$).

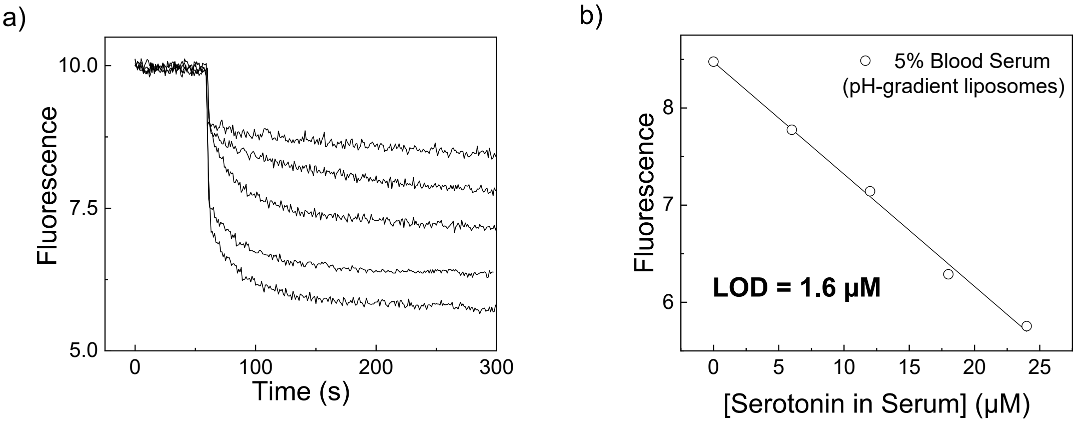

Figure S12. a) Fluorescence changes of POPC/POPS⊃CB8/MDAP liposomes ($pH_{out} = 10.8$, $pH_{in} = 3.5$) upon addition of 5% human blood serum spiked with varying concentrations of serotonin. b) Plot of the final fluorescence at ca. 300 s against the serotonin concentration in the spiked blood serum samples. The limit of detection (LOD) of serotonin was calculated from the slope and the triple standard deviation of the blank of the linear calibration curve ($R^2 =$ 0.99).

CB7/BE Reporter Pair

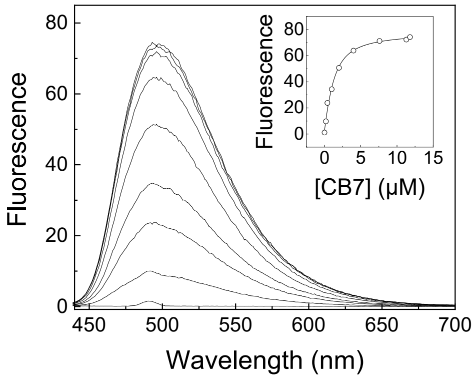

Figure S13. Binding constant determination of BE with CB7 in 100 mM sodium citrate buffer at pH 3.5 by fluorescence titration (λ_{ex} = 425 nm and λ_{em} = 495 nm) using 1 µM BE, (K_a = (1.3 ± 0.3) × 10^6 M^{-1}).

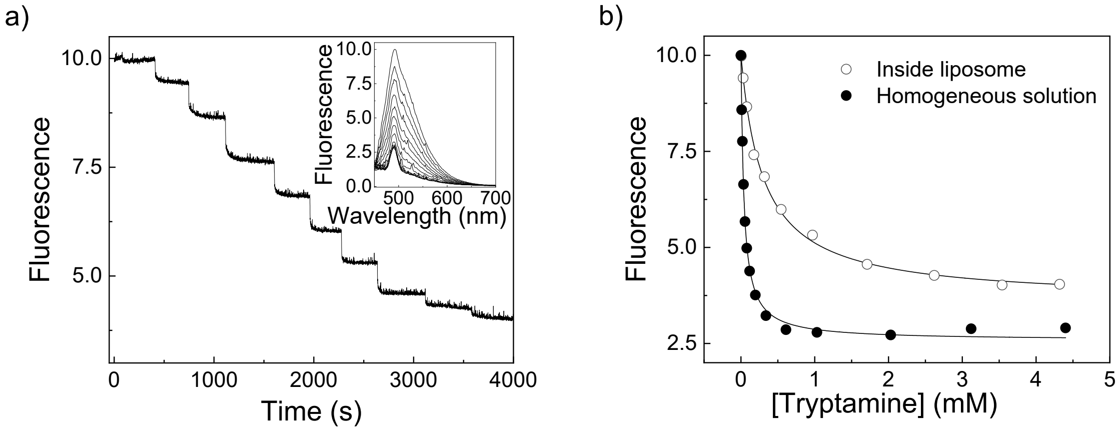

Figure S14. a) Time-dependent fluorescence changes of POPC/POPS⊃CB7/BE liposomes (pH_{out} = 10.8, pH_{in} = 3.5) during successive addition of tryptamine. The inset shows the fluorescence spectral changes during a conventional fluorescence titration with tryptamine and CB7/BE (2.2 µM CB7; 3.8 µM BE) in 100 mM sodium citrate, pH 3.5. b) Respective titration curves (λ_{ex} = 420 nm, and λ_{em} = 495 nm).

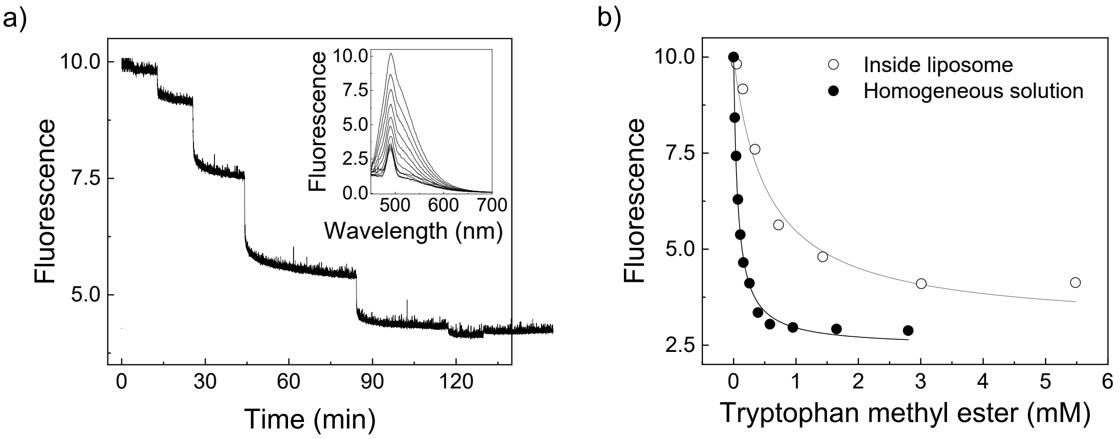

Figure S15. a) Time-dependent fluorescence changes of POPC/POPS⊃CB7/BE liposomes (pH_{out} = 10.8, pH_{in} = 3.5) during successive addition of tryptophan methyl ester. The inset shows the fluorescence spectral changes during a conventional fluorescence titration with tryptophan methyl ester and CB7/BE (2.2 µM CB7; 3.8 µM BE) in 100 mM sodium citrate, pH 3.5. b) Respective titration curves (λ_{ex} = 420 nm, and λ_{em} = 495 nm).

Table S2. Binding constant values of analytes with reporter pair CB7/BE.

Analyte	K_{app} (M^{-1}) pH gradient 3.5–10.8	K_a (M^{-1}) Homogeneous solution
Tryptamine	$(5.7 \pm 0.3) \times 10^3$	$(4.5 \pm 0.3) \times 10^4$
Tryptophan methyl ester	$(3.1 \pm 0.4) \times 10^3$	$(2.5 \pm 0.2) \times 10^4$

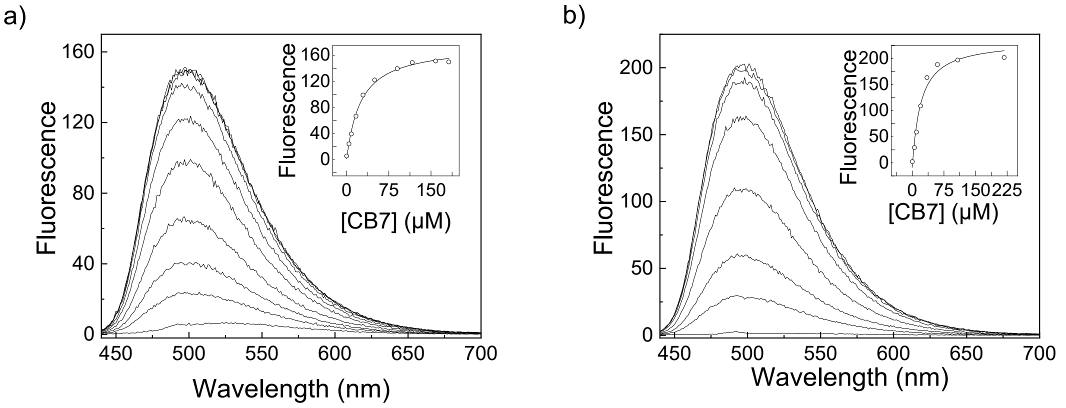

Figure S16. Fluorescence titration of 2 μM PLM with CB7. a) In 100 mM NaH_2PO_4, pH 7.5. The inset shows the corresponding titration plot with fitted line ($K_a = (4.2 \pm 0.4) \times 10^4$ M^{-1}). b) In 100 mM sodium citrate buffer, pH 3.5. The inset shows the corresponding titration plot with fitted line ($K_a = (5.3 \pm 1.0) \times 10^4$ M^{-1}), (λ_{ex} = 425 nm and λ_{em} = 495 nm).

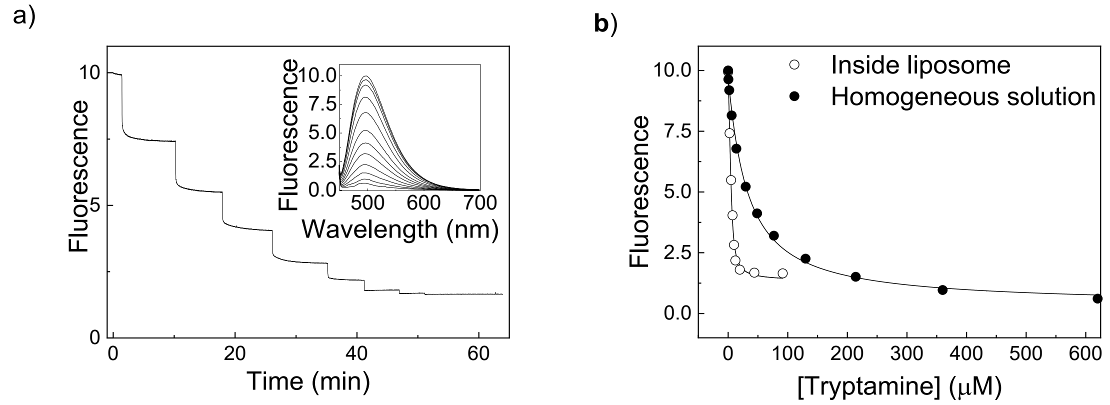

Figure S17. a) Time-dependent fluorescence changes of POPC/POPS⊃CB7/PLM liposomes (pH_{out} = 10.8, pH_{in} = 3.5) during successive addition of tryptamine. The inset shows the fluorescence spectral changes during a conventional fluorescence titration with tryptamine and CB7/PLM (0.5 μM CB7; 0.7 μM PLM) in 100 mM sodium citrate, pH 3.5. b) Respective titration curves (λ_{ex} = 425 nm, and λ_{em} = 495 nm).

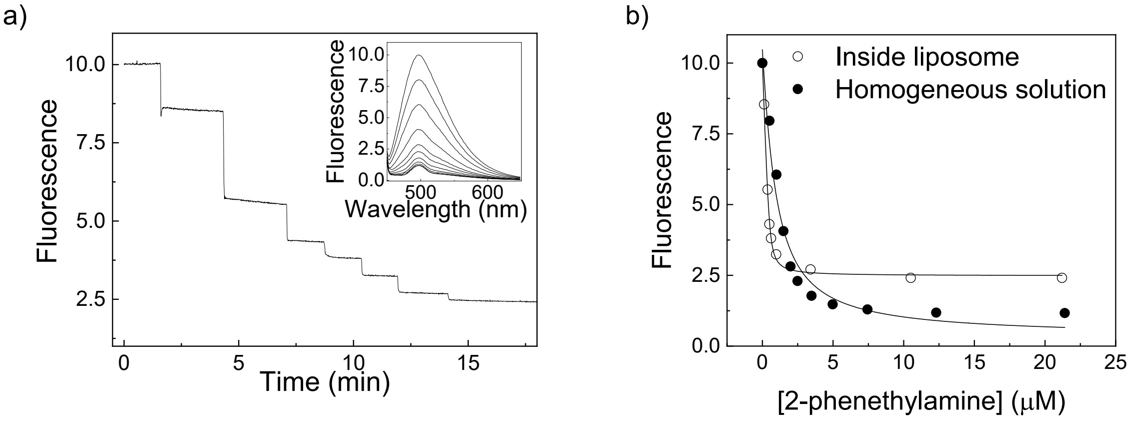

Figure S18. a) Time-dependent fluorescence changes of POPC/POPS⊃CB7/PLM liposomes (pH_{out} = 10.8, pH_{in} = 3.5) during successive addition of 2-phenethylamine. The inset shows the fluorescence spectral changes during a conventional fluorescence titration with 2-phenethylamine and CB7/PLM (0.5 μM CB7; 0.7 μM PLM) in 100 mM sodium citrate, pH 3.5. b) Respective titration curves (λ_{ex} = 425 nm, and λ_{em} = 495 nm).

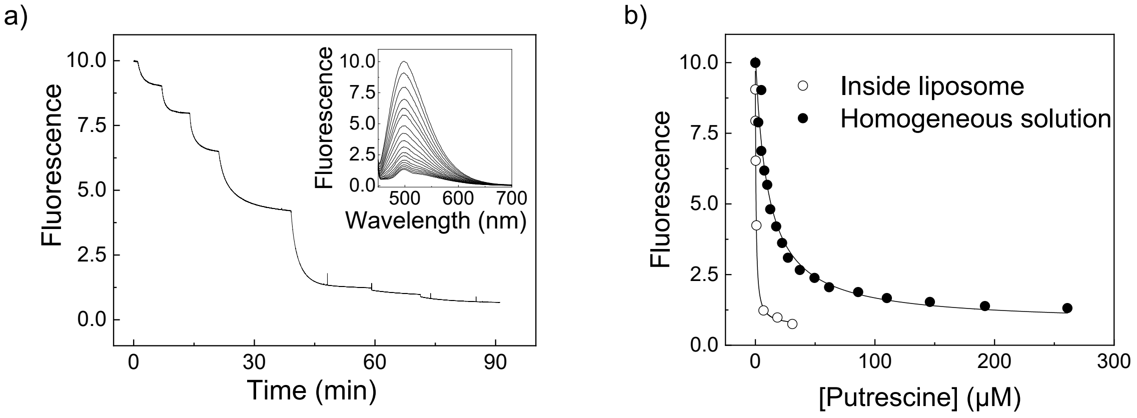

Figure S19. a) Time-dependent fluorescence changes of POPC/POPS⊃CB7/PLM liposomes (pH_{out} = 10.8, pH_{in} = 3.5) during successive addition of putrescine. The inset shows the fluorescence spectral changes during a conventional fluorescence titration with putrescine and CB7/PLM (0.5 μM CB7; 0.7 μM PLM) in 100 mM sodium citrate, pH 3.5. b) Respective titration curves (λ_{ex} = 425 nm, and λ_{em} = 495 nm).

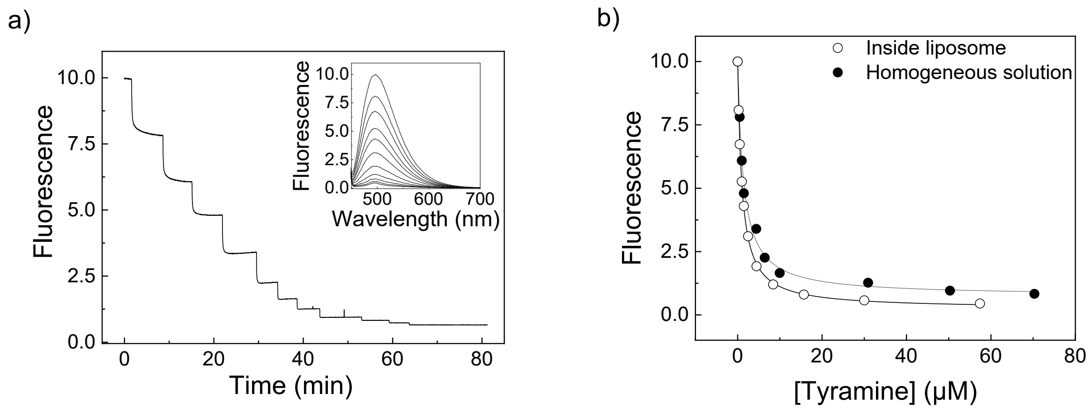

Figure S20. a) Time-dependent fluorescence changes of POPC/POPS⊃CB7/PLM liposomes (pH_{out} = 10.8, pH_{in} = 3.5) during successive addition of tyramine. The inset shows the fluorescence spectral changes during a conventional fluorescence titration with tyramine and CB7/PLM (0.5 μM CB7; 0.7 μM PLM) in 100 mM sodium citrate, pH 3.5. b) Respective titration curves (λ_{ex} = 425 nm, and λ_{em} = 495 nm).

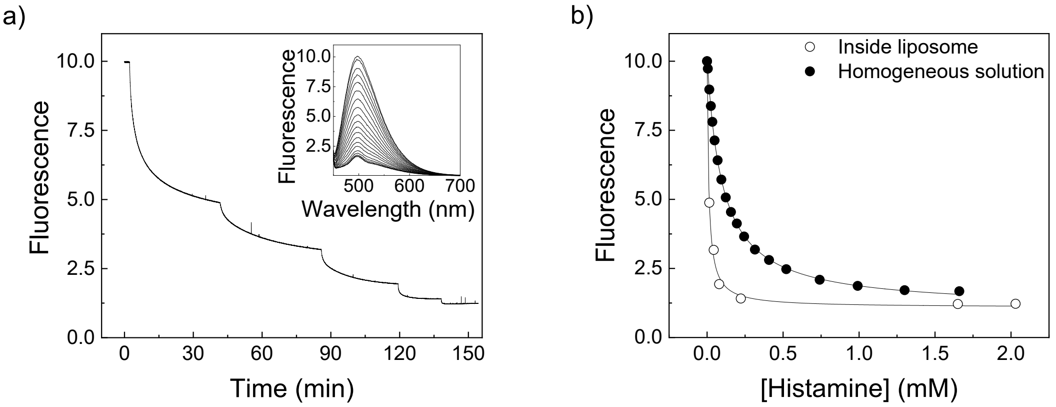

Figure S21. a) Time-dependent fluorescence changes of POPC/POPS⊃CB7/PLM liposomes (pH_{out} = 10.8, pH_{in} = 3.5) during successive addition of histamine. The inset shows the fluorescence spectral changes during a conventional fluorescence titration with histamine and CB7/PLM (0.5 μM CB7; 0.7 μM PLM) in 100 mM sodium citrate, pH 3.5. b) Respective titration curves (λ_{ex} = 425 nm, and λ_{em} = 495 nm).

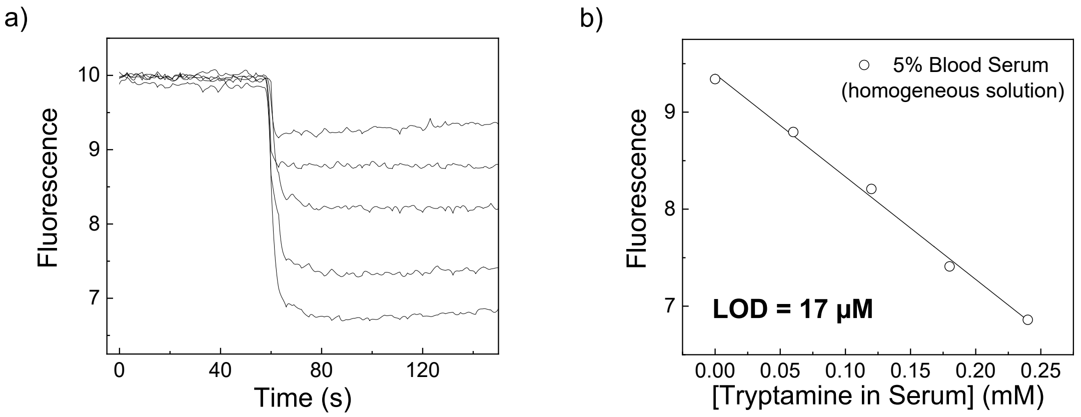

Figure S22. a) Time dependence of fluorescence changes of CB7/PLM upon addition of tryptamine with 5% human blood serum (CB7 0.5 µM and PLM 0.7 µM) in 100 mM sodium citrate, pH 3.5 b) The limit of detection (LOD) of tryptamine was calculated using linear calibration curve ($R^2 = 0.99$) afforded the LOD of 17 µM.

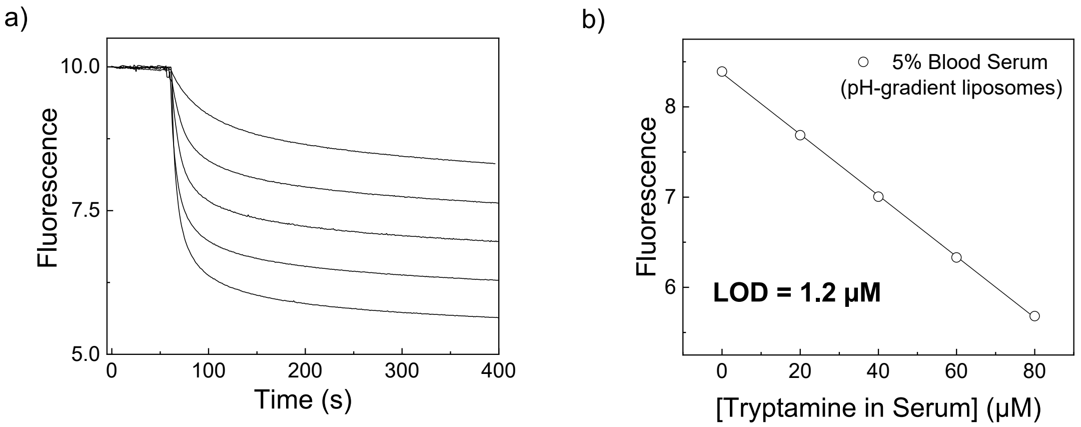

Figure S23. a) Time dependence of fluorescence changes upon addition of tryptamine with 5% human blood serum *via* the pH gradient (aqueous interior of the liposome 100 mM sodium citrate, pH 3.5 and exterior of the liposome 100 mM Na_2HPO_4, pH 10.8) across the POPC/POPS⊃CB7/PLM liposomes. b) The limit of detection (LOD) of tryptamine was calculated using linear calibration curve ($R^2 = 0.99$) afforded the LOD of 1.2 µM.

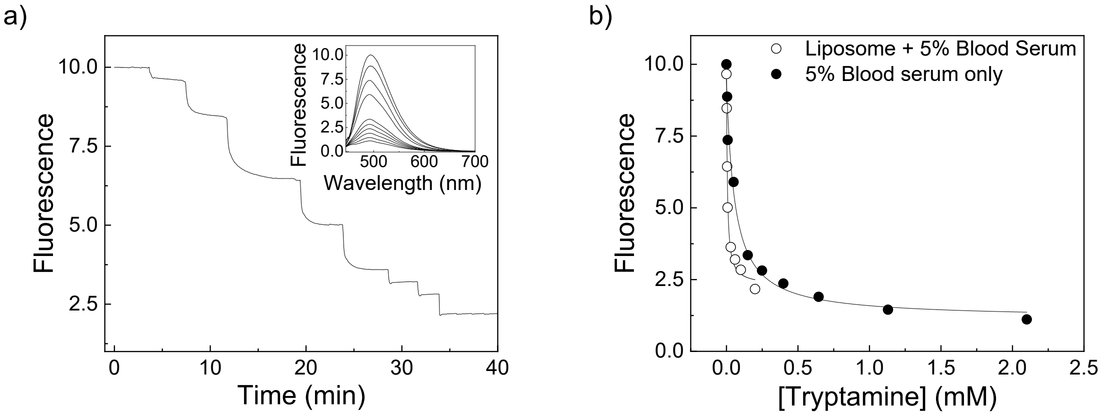

Figure S24. a) Time-dependent fluorescence changes of POPC/POPS⊃CB7/PLM liposomes (pH_{out} = 10.8, pH_{in} = 3.5) during successive addition of tryptamine. The inset shows the fluorescence spectral changes during a conventional fluorescence titration with tryptamine and CB7/PLM (0.5 μM CB7; 0.7 μM PLM) in 100 mM sodium citrate, pH 3.5, 5% blood serum b) Respective titration curves (λ_{ex} = 425 nm, and λ_{em} = 495 nm).

HP-β-CD/BE Reporter Pair

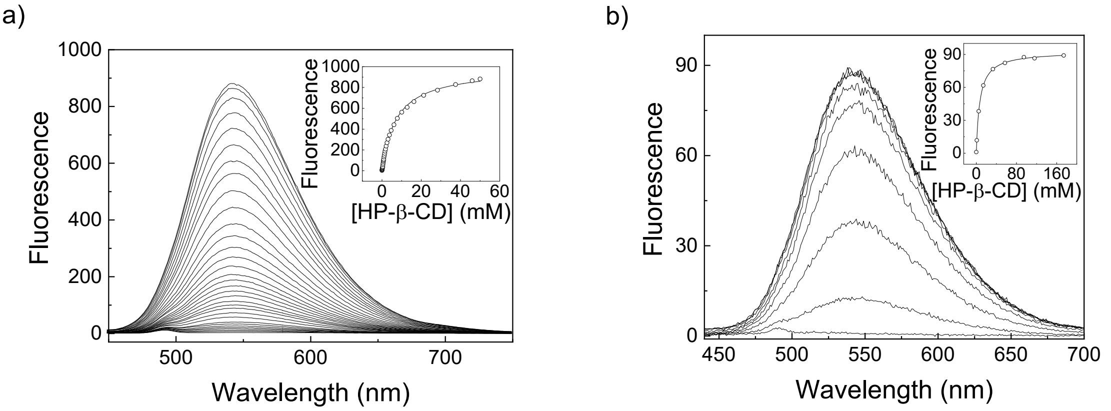

Figure S25. a) Fluorescence titration of 5 μM berberine with HP-*β*-CD in 100 mM NaH_2PO_4, pH 7.5. The inset shows the corresponding titration plot with fitted line (K_a = (137 ± 4.0) M^{-1}). b) Fluorescence titration of 4 μM berberine with HP-*β*-CD in sodium citrate, pH 3.5. The inset shows the corresponding titration plot with fitted line (K_a = (141 ± 4.0) M^{-1}), (λ_{ex} = 420 nm, and λ_{em} = 540 nm).

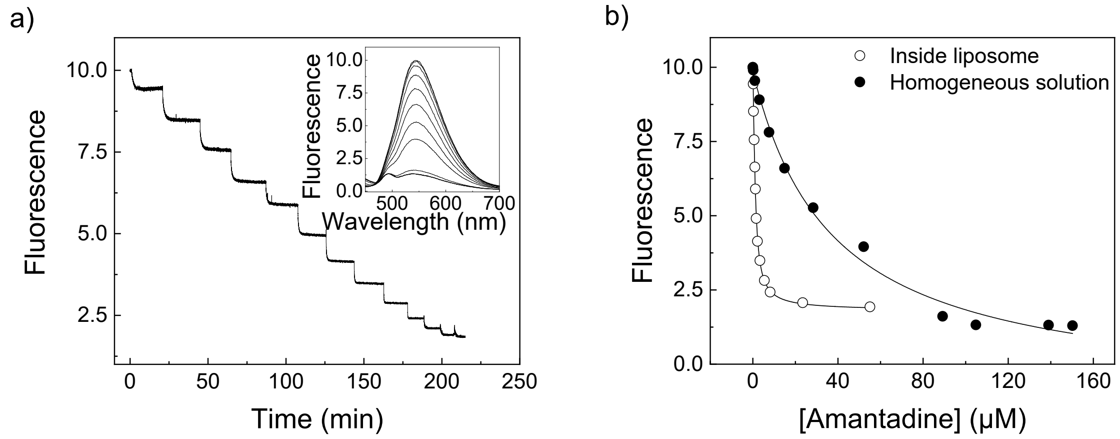

Figure S26. a) Time-dependent fluorescence changes of POPC/POPS⊃HP-*β*-CD/BE liposomes (pH_{out} = 10.8, pH_{in} = 3.5) during successive addition of amantadine. The inset shows the fluorescence spectral changes during a conventional fluorescence titration with amantadine and HP-*β*-CD/BE (4.2 μM HP-*β*-CD; 5 μM BE) in 100 mM sodium citrate, pH 3.5. b) Respective titration curves (λ_{ex} = 420 nm, and λ_{em} = 540 nm).

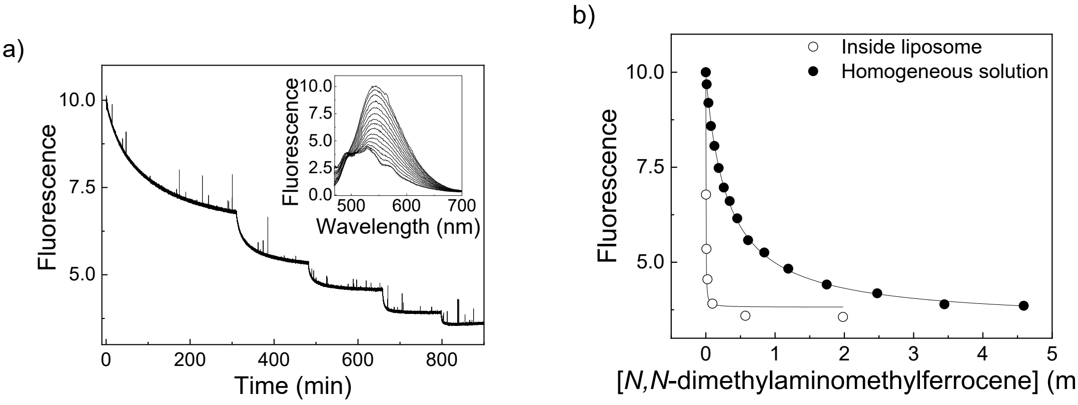

Figure S27. a) Time-dependent fluorescence changes of POPC/POPS⊃HP-*β*-CD/BE liposomes (pH_{out} = 10.8, pH_{in} = 3.5) during successive addition of *N,N*-dimethylaminomethylferrocene. The inset shows the fluorescence spectral changes during a conventional fluorescence titration with *N,N*-dimethylaminomethylferrocene and HP-*β*-CD/BE (4.2 µM HP-*β*-CD; 5 µM BE) in 100 mM sodium citrate, pH 3.5. b) Respective titration curves (λ_{ex} = 420 nm, and λ_{em} = 540 nm).

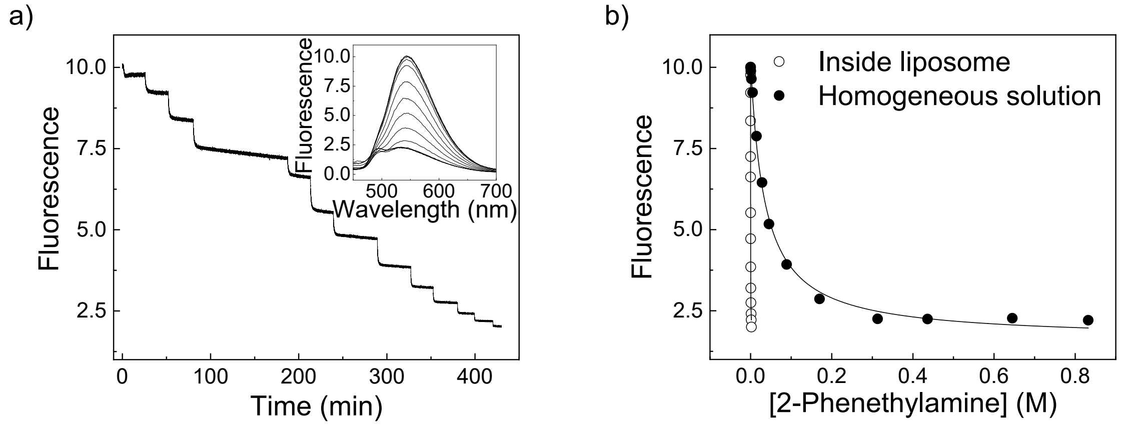

Figure S28. a) Time-dependent fluorescence changes of POPC/POPS⊃HP-*β*-CD/BE liposomes (pH_{out} = 10.8, pH_{in} = 3.5) during successive addition of 2-phenethylamine. The inset shows the fluorescence spectral changes during a conventional fluorescence titration with 2-phenethylamine and HP-*β*-CD/BE (4.2 µM HP-*β*-CD; 5 µM BE) in 100 mM sodium citrate, pH 3.5. b) Respective titration curves (λ_{ex} = 420 nm, and λ_{em} = 540 nm).

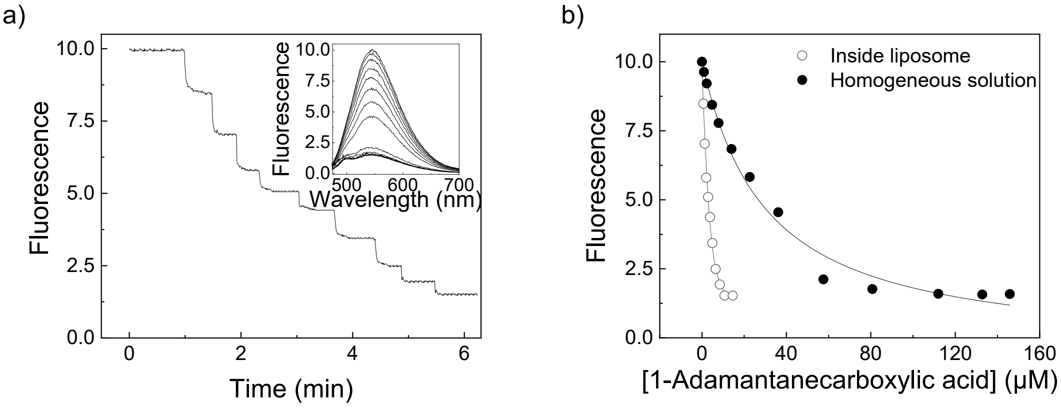

Figure S29. a) Time-dependent fluorescence changes of POPC/POPS⊃HP-*β*-CD/BE liposomes (pH_{out} = 3.0, pH_{in} = 10.8) during successive addition of 1-adamantanecarboxylic acid. The inset shows the fluorescence spectral changes during a conventional fluorescence titration with 1-adamantanecarboxylic acid and HP-*β*-CD/BE (3.8 μM HP-*β*-CD; 4 μM BE) in 100 mM Na_2HPO_4, pH 10.8. b) Respective titration curves (λ_{ex} = 420 nm, and λ_{em} = 540 nm).

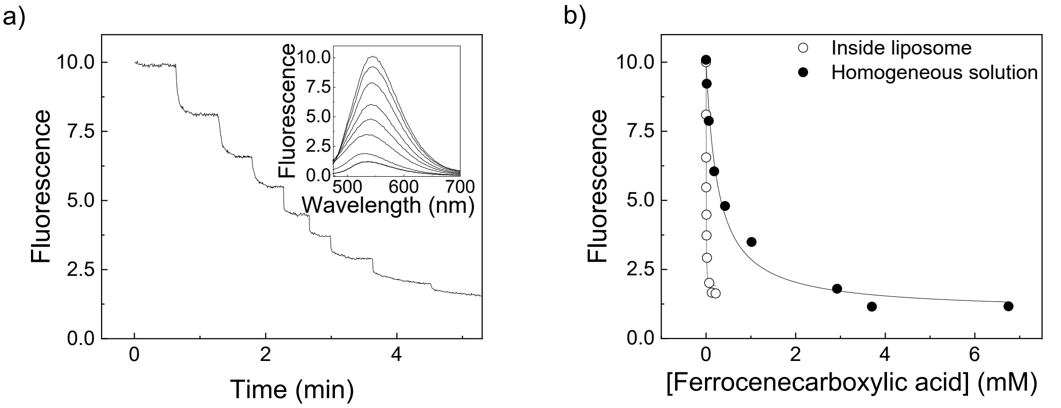

Figure S30. a) Time-dependent fluorescence changes of POPC/POPS⊃HP-*β*-CD/BE liposomes (pH_{out} = 3.0, pH_{in} = 10.8) during successive addition of ferrocenecarboxylic acid. The inset shows the fluorescence spectral changes during a conventional fluorescence titration with ferrocenecarboxylic acid and HP-*β*-CD/BE (4.9 μM HP-*β*-CD; 5 μM BE) in 100 mM Na_2HPO_4, pH 10.8. b) Respective titration curves (λ_{ex} = 420 nm, and λ_{em} = 540 nm).

Table S3. Analyte protonation and deprotonation species in different pH.

Analyte	pK_a	Ref. for pK_a	Base form	Acid form	[B]/[BH$^+$])[a]	
					pH 3.5	pH 10.8
L-Tryptophanamide	7.5	[5]	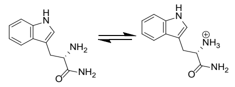		<1:100	>100:1
Tryptamine	9.3	[5]	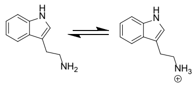		<1:100	32:1
L-Tryptophan methyl ester	7.6	[5]	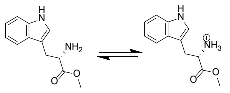		<1:100	>100:1
Serotonin	9.9	[6]	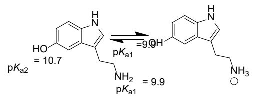		<1:100	8:1
	10.7	[6]	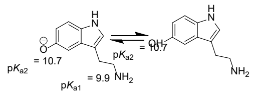		<1:100	~1:1
Tyramine	9.7	[7]	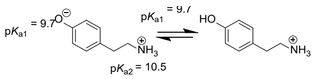		<1:100	13:1
	10.5	[7]	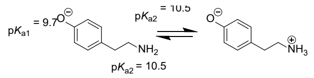		<1:100	2:1
Putrescine	9.3	[8]	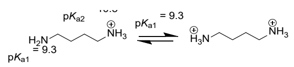		<1:100	32:1
	10.5	[8]	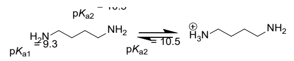		<1:100	2:1
Amantadine	10.8	[9]	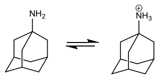		<1:100	1:1
Histamine	6.0	[10]	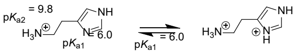		<1:100	>100:1
	9.8	[10]	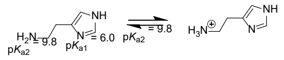		<1:100	10:1
Phenethylamine	9.8	[11]	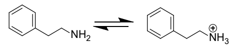		<1:100	10:1
1-Adamantanecarboxylic acid	5.1	[12]	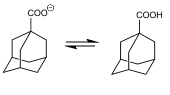		<1:100[b]	>100:1
Ferrocenecarboxylic acid	4.2	[13]	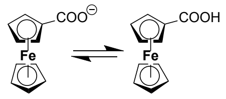		<1:100[b]	>100:1
N,*N*-Dimethylaminomethyl-ferrocene	8.8	[14]	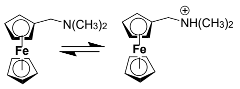		<1:100	100:1

[a] Calculated using the Henderson-Hasselbalch equation. [b] Calculated for pH 3.0.

Buffer Capacity

Buffer Capacity in Homogeneous Solution

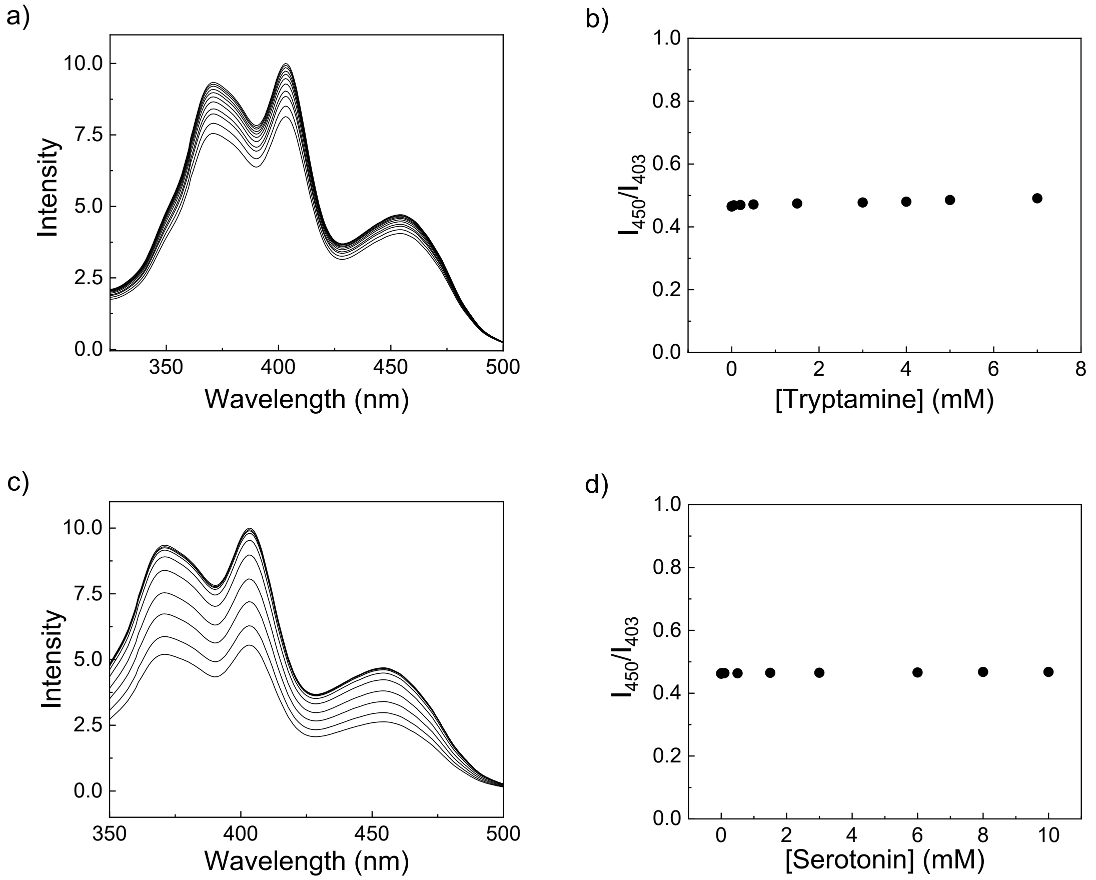

Figure S31. Excitation spectra (λ_{em} = 511 nm) of HPTS-encapsulated POPC/POPS liposomes (inside: 100 mM NaH_2PO_4, 1 mM HPTS, pH 7.2; outside: 100 mM Na_2HPO_4, pH 10.8) upon addition of a) tryptamine or c) serotonin *via* the pH gradient. Ratiometric signal of HPTS as a function of the concentration of b) tryptamine and d) serotonin.

Buffer Capacity in Blood Serum Samples

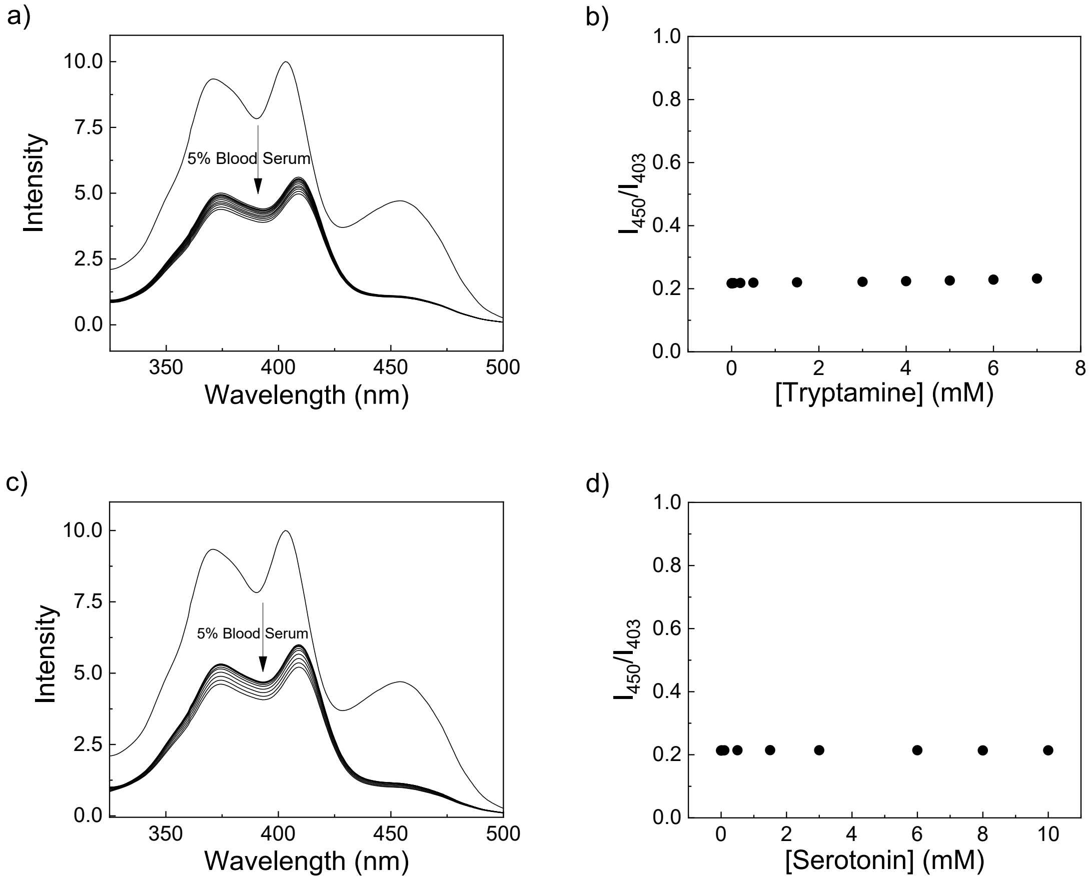

Figure S32. Excitation spectra (λ_{em} = 511 nm) of HPTS-encapsulated POPC/POPS liposomes (inside: 100 mM NaH_2PO_4, 1 mM HPTS, pH 7.2; outside: 100 mM Na_2HPO_4, pH 10.8) upon addition of 5% blood serum and subsequent addition of a) tryptamine and c) serotonin. Ratiometric signal of the HPTS-encapsulated POPC/POPS liposomes in 5% blood serum as a function of the concentration of b) tryptamine and d) serotonin.

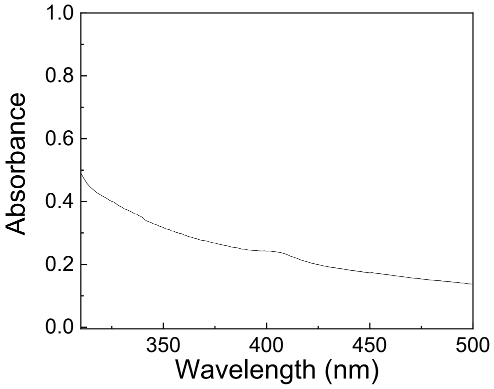

Figure S33. Absorption spectrum of 5% blood serum in 100 mM sodium phosphate buffer, pH 10.8.

References

[1] a) C. Marquez, H. Fang, W. M. Nau, *IEEE Trans. Nanobioscience* **2004**, *3*, 39-45; b) J. Kim, I.-S. Jung, S.-Y. Kim, E. Lee, J.-K. Kang, S. Sakamoto, K. Yamaguchi, K. Kim, *J. Am. Chem. Soc.* **2000**, *122*, 540-541.

[2] a) A. J. Blacker, J. Jazwinski, J.-M. Lehn, *Helv. Chim. Acta* **1987**, *70*, 1-12; b) V. Sindelar, M. A. Cejas, F. M. Raymo, W. Chen, S. E. Parker, A. E. Kaifer, *Eur. J. Chem.* **2005**, *11*, 7054-7059.

[3] R. Hein, C. B. Uzundal, A. Hennig, *Org. Biomol. Chem.* **2016**, *14*, 2182-2185.

[4] a) F. Biedermann, G. Ghale, A. Hennig, W. M. Nau, *Commun. Biol* **2020**, *3*, 383; b) A. Hennig, A. Hoffmann, H. Borcherding, T. Thiele, U. Schedler, U. Resch-Genger, *Chem. Commun.* **2011**, *47*, 7842-7844.

[5] S.-N. Chen, M. Z. Hoffman, *J. Phys. Chem.* **1974**, *78*, 2099-2102.

[6] J. Pratuangdejkul, W. Nosoongnoen, G.-A. Guérin, S. Loric, M. Conti, J.-M. Launay, P. Manivet, *Chem. Phys. Lett.* **2006**, *420*, 538-544.

[7] T. Kappe, M. D. Armstrong, *J. Med. Chem.* **1965**, *8*, 368-374.

[8] J. G. Voet, E. C. Andersen, *Arch. Biochem. Biophys.* **1984**, *233*, 88-92.

[9] a) C. Wang, K. Takeuchi, L. H. Pinto, R. A. Lamb, *J. Virol.* **1993**, *67*, 5585-5594; b) W. Danysz, A. Dekundy, A. Scheschonka, P. Riederer, *J. Neural Transm.* **2021**, *128*, 127-169.

[10] C. R. Ganellin, *J. Pharm. Pharmacol.* **1973**, *25*, 787-792.

[11] S. J. Gluck, J. A. Cleveland, *J. Chromatogr. A* **1994**, *680*, 43-48.

[12] W. Cullen, S. Turega, C. A. Hunter, M. D. Ward, *Chem. Sci.* **2015**, *6*, 625-631.

[13] T. Skeika, C. R. Zuconelli, S. T. Fujiwara, C. A. Pessoa, *Sensors* **2011**, *11*, 1361-1374.

[14] N. W. Duffy, J. Harper, P. Ramani, R. Ranatunge-Bandarage, B. H. Robinson, J. Simpson, *J. Organomet. Chem.* **1998**, *564*, 125-131.
